# The Growth Mechanism of Transition Metal Dichalcogenides by using Sulfurization of Pre-deposited Transition Metals and the 2D Crystal Hetero-structure Establishment

**DOI:** 10.1038/srep42146

**Published:** 2017-02-08

**Authors:** Chong-Rong Wu, Xiang-Rui Chang, Chao-Hsin Wu, Shih-Yen Lin

**Affiliations:** 1 Graduate Institute of Electronics Engineering, National Taiwan University, No. 1, Sec. 4, Roosevelt Rd., Taipei 10617, Taiwan; 2 Research Center for Applied Sciences, Academia Sinica, No. 128, Sec. 2, Academia Rd., Taipei 11529, Taiwan

## Abstract

A growth model is proposed for the large-area and uniform MoS_2_ film grown by using sulfurization of pre-deposited Mo films on sapphire substrates. During the sulfurization procedure, the competition between the two mechanisms of the Mo oxide segregation to form small clusters and the sulfurization reaction to form planar MoS_2_ film is determined by the amount of background sulfur. Small Mo oxide clusters are observed under the sulfur deficient condition, while large-area and complete MoS_2_ films are obtained under the sulfur sufficient condition. Precise layer number controllability is also achieved by controlling the pre-deposited Mo film thicknesses. The drain currents in positive dependence on the layer numbers of the MoS_2_ transistors with 1-, 3- and 5- layer MoS_2_ have demonstrated small variation in material characteristics between each MoS_2_ layer prepared by using this growth technique. By sequential transition metal deposition and sulfurization procedures, a WS_2_/MoS_2_/WS_2_ double hetero-structure is demonstrated. Large-area growth, layer number controllability and the possibility of hetero-structure establishment by using sequential metal deposition and following sulfurization procedures have revealed the potential of this growth technique for practical applications.

With the advantage of theoretically predicated ultra-high carrier mobility values, graphene is believed to be of potential application for high-speed electronics in the below 10 nm technology node^1^,^2^. However, due to its zero-bandgap nature, graphene transistors usually exhibited low ON/OFF ratios. This disadvantage has substantially limited the practical application of graphene for logic circuits. To overcome this disadvantage of graphene, people have gradually turned their focus to other two-dimensional (2D) crystals with bandgaps such as transition metal dichalcogenides (TMDs) in recent years^3^,^4^,^5^.

One of the promising TMDs for the transistor application is Molybdenum disulfide (MoS_2_)^6^,^7^. Although its mobility value is much lower than graphene, MoS_2_ transistors with high ON/OFF ratios around 10^8^ have already been demonstrated, which has revealed the potential of MoS_2_ for the transistor application^8^,^9^,^10^. One promising approach to obtain this material is the traditional mechanical exfoliation^11^. Although MoS_2_ flakes with high crystalline quality can be obtained by using this method, uniform and large-area MoS_2_ films are still required for the practical application. In nowadays, the mainstream method to grow large-area TMD films is the chemical vapor deposition (CVD). By using either MoO_3_ or MoCl_5_ as the precursor, MoS_2_ films can be synthesized in hot furnace with the co-evaporation of sulfur (S) powder at 700–850 °C^12^,^13^. With graphene/sapphire samples as the substrates, MoS_2_/graphene hetero-structures have also been demonstrated prepared by CVD for device applications^14^.

Although CVD is very promising for large-area MoS_2_ growth, there are still several limitations for this technique. Since the transition metal precursor and the S powder are uniformly distributed onto the substrates at high growth temperature, selective growth is difficult to achieve with the traditional CVD growth technique. In this case, when dealing with more complicated 2D crystal hetero-structures, selective etching to expose contact areas for electrodes would become a necessary processing procedure for device fabrications. Until now, there is no such report regarding selective etching of 2D crystals in literature. On the other hand, for the preparation of different TMDs to establish 2D crystal hetero-structures, different precursors have to be adopted. In this case, growth optimizations are required for each 2D crystal, which will further complicate the growth procedure of the 2D crystal hetero-structure. Therefore, if a standard growth procedure can be applied to different TMD growth, the establishment of 2D crystal hetero-structures would become much easier and more promising for practical applications^15^. In previous publications, it has been demonstrated that by sulfurizing pre-deposited Mo films in hot furnace, large-area MoS_2_ films can be grown on sapphire substrates^16^,^17^,^18^,^19^. By using the similar approach, large-area WS_2_ can also be prepared^19^,^20^. Therefore, we believe that by repeating the sequential transition metal deposition and the sulfurization procedures, large-area and uniform TMD hetero-structures may be prepared. To achieve this goal, further investigation over the growth mechanisms of sulfurizing pre-deposited transition metal films in hot furnace is required.

In this paper, we have investigated the growth mechanisms of large-area MoS_2_ films by sulfurizing pre-deposited Mo thin films at 750 °C. By fixing the thickness of Mo films at 1 nm and decreasing the sulfur supply, similar Raman peak differences are observed. The results suggest that the same MoS_2_ layer numbers are obtained for samples grown under different amounts of sulfur powder. The picture obtained by using cross-sectional high-resolution transmission electron microscopy (HRTEM) is adopted to investigate the actual layer numbers and film morphologies of the MoS_2_ samples. With the observation of small Mo oxide clusters on the surface for the sample grown under the sulfur deficient condition, a growth model is proposed to explain the growth mechanisms of the MoS_2_ films by using the sulfurization of pre-deposited Mo films. By sequential transition metal deposition and sulfurization procedures, we have also demonstrated a WS_2_/MoS_2_/WS_2_ double hetero-structure grown through this growth method.

## Results and Discussions

### Characterizations of MoS_2_ grown by using different amounts of sulfur powder

**Figure 1:**
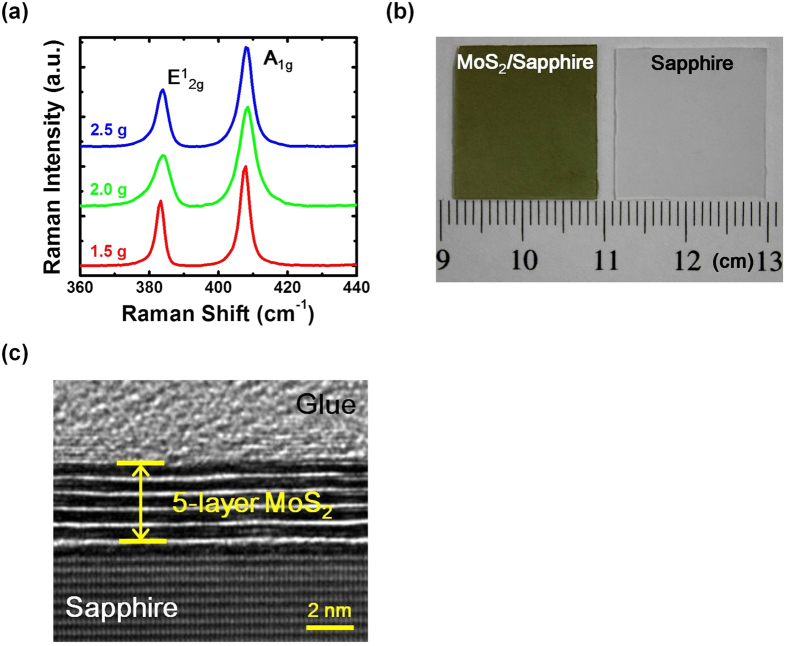
(**a**) The Raman spectrums of the MoS_2_ samples prepared by using sulfurization of 1.0 nm Mo with 2.5, 2.0 and 1.5 g sulfur powder. (**b**) The picture and (**c**) the cross-sectional HRTEM image of the sample grown with 1.5 g sulfur powder. A blank sapphire substrate is also shown in (**b**) for comparison.

The Raman spectra of the MoS_2_ samples prepared by using sulfurization of 1.0 nm Mo with 2.5, 2.0 and 1.5 g sulfur powder are shown in Fig. 1(a). As shown in the figure, two characteristic Raman peaks are observed for the three samples with one peak 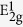 located at 384 cm^−1^ and the other A_1g_ at 408 cm^−1^. They are associated with the in-plane and out-of-plane vibration modes of the MoS_2_ crystal, respectively^21^. It has also been reported that the frequency difference between the two peaks would increase from ~21 cm^−1^ to >24 cm^−1^ for single-layer to >4-layer MoS_2_ films^13^. The Raman peak differences *∆k* of the three samples grown by using sulfur powder 2.5, 2 and 1.5 g are 24.14, 24.37 and 24.50 cm^−1^, respectively. The results suggest that although different amounts of sulfur powder are adopted, similar MoS_2_ layer numbers around 4–5 layers are obtained for the three samples. The phenomenon also indicates that the layer numbers of the MoS_2_ films prepared through this approach is determined by the pre-deposited Mo film thicknesses. It has been proposed in previous publications that MoS_2_ films with better crystalline quality would result in reduced 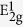 full width at the half maximum (FWHM) value and enhanced 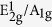 peak ratios^12^,^22^. The FWHM values of the 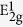 peak are 3.55, 3.4 and 2.48 cm^−1^, while the 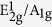 peak intensity ratios are 0.57, 0.51 and 0.65 for the three samples, respectively. The smallest 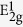 FWHM value and the highest 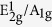 peak intensity ratio are observed for the sample prepared by using 1.5 g sulfur power. The results suggest that 1.5 g sulfur is the optimized growth parameter for the sulfurization of 1.0 nm Mo film. The picture of the sample sulfurized by using 1.5 g sulfur power is shown in Fig. 1(b). A blank sapphire substrate is also shown in the figure for comparison. The picture has revealed a uniform and large-area MoS_2_ film grown on the sapphire substrate, which has demonstrated the potential of this technique for wafer-scale TMD growth. To confirm the layer number of the MoS_2_ film, the cross-sectional HRTEM image of the sample grown by using 1.5 g sulfur powder is shown in Fig. 1(c). As shown in the figure, 5-layer MoS_2_ is clearly observed for the sample. The results are consistent with the observation obtained from the Raman spectra.

### The MoS_2_ film grown under the sulfur deficient condition

**Figure 2:**
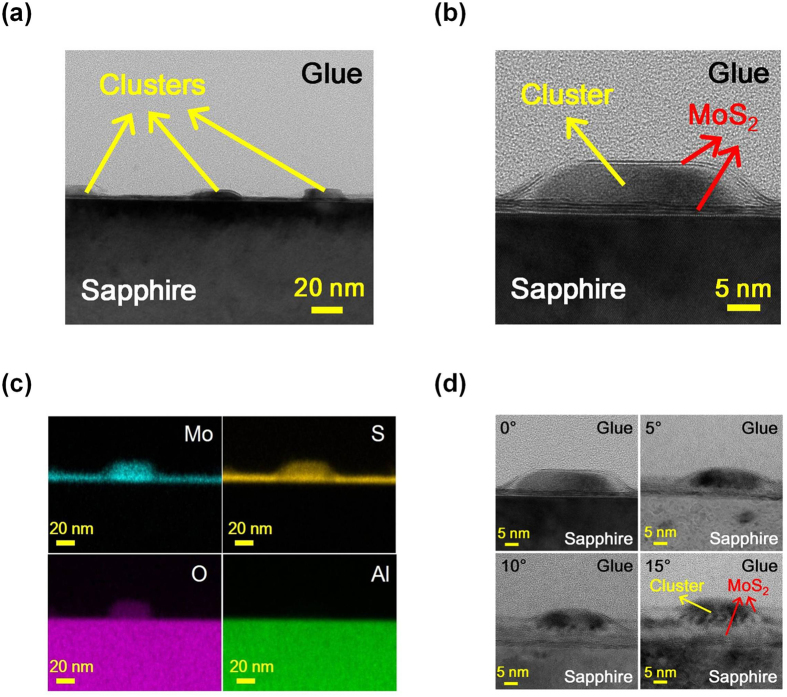
The cross-sectional HRTEM images of the sample prepared with no sulfur supply under (**a**) lower and (**b**) higher magnifications. (**c**) HAADF mappings of elements Mo, S, O and Al and (**d**) the HRTEM images of the same sample with 0°, 5°, 10° and 15° tilt angles from the cross-sectional view.

To further investigate the growth mechanism for the sulfurization of pre-deposited Mo films, an additional sample with no sulfur supply is prepared for comparison. Since there is always residue sulfur accumulation near the downstream of the growth chamber after repeating growth cycles, it is expected that a small amount of sulfur will still diffuse to the sample surface and result in MoS_2_ growth. However, under such a sulfur deficient condition, not all the pre-deposited Mo will be transformed into MoS_2_. The cross-sectional HRTEM image of the sample prepared with no sulfur supply is shown in Fig. 2(a). As shown in the figure, clusters of materials instead of flat 2D crystal films spreading over the sample surface are observed. To further investigate this phenomenon, the HRTEM image with a higher magnification of the same sample is shown in Fig. 2(b). It seems that the sample surface including the small clusters is still covered by few-layer MoS_2_. To verify the chemical compositions of the small clusters, high-angle annular dark field (HAADF) mappings of elements Mo, sulfur (S), oxygen (O) and Aluminum (Al) are shown in Fig. 2(c). The mapping picture of Al would indicate the location of the sapphire substrate. As shown in the figure, besides a thin layer of the Mo and S signals observed above the sapphire substrate, the signals of the two elements are also observed on the cluster. The results may suggest that few-layer MoS_2_ fully cover the sample surface including the clusters. The phenomenon is consistent with the observation of the cross-sectional HRTEM image shown in Fig. 2(b). On the other hand, beside the sapphire substrate, the O signal is also observed on the clusters. Since the cluster may be fully covered with few-layer MoS_2_, it is possible that the S signal observed on the clusters comes from the covering few-layer MoS_2_ films. In this case, it is reasonable to assume that the clusters contain mostly Mo oxides. The other phenomenon observed in Fig. 2(b) is that there seems to be MoS_2_ films underneath the small Mo oxide clusters. To verify this phenomenon, the cross-sectional HRTEM images of the sample with 0°, 5°, 10° and 15° tilt angles from the cross-sectional view are shown in Fig. 2(d). It is clearly seen from the figures that the Mo oxide clusters are covered with few-layer MoS_2_. In this case, the observation of MoS_2_ film underneath the Mo oxide clusters observed in Fig. 2(b) should actually come from the flattened few-layer MoS_2_ film in front of the Mo oxide clusters. The results of few-layer MoS_2_ covering the whole sample surface including the Mo oxide clusters suggest that the Mo oxide clusters should form before the formation of the few-layer MoS_2_ file.

### The growth mechanism of MoS_2_ by using sulfurization of pre-deposited Mo films

**Figure 3:**
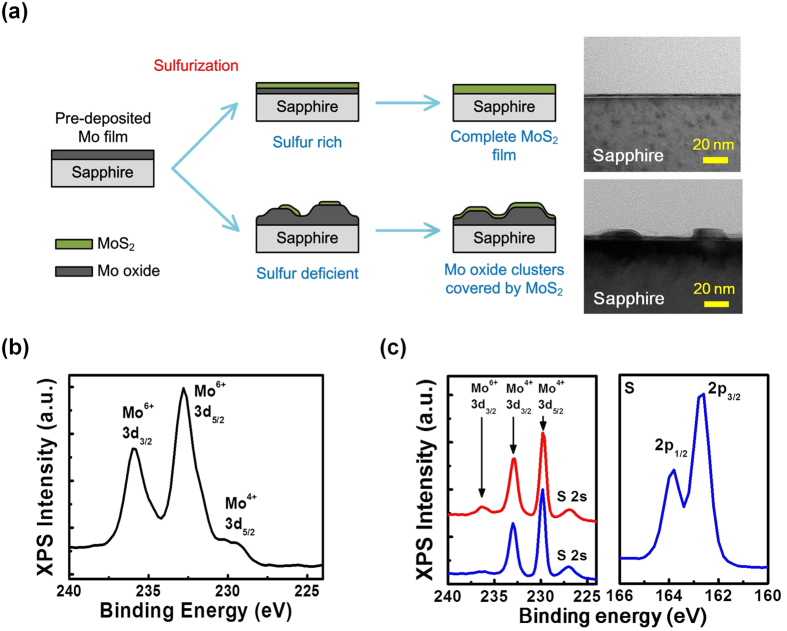
(**a**) The schematic diagrams showing the growth evolution of the samples prepared under sulfur sufficient and deficient conditions. (**b**) The XPS curve measured before the sulfurization procedure. (**c**) The XPS curves measured after the sulfurization procedure for the two samples grown under sulfur sufficient (1.5 g sulfur, blue curve) and deficient (no sulfur supply, red curve) conditions, respectively.

With the results discussed above, a possible growth model for the MoS_2_ samples prepared by using sulfurization of pre-deposited Mo films is proposed. The schematic diagrams showing the growth evolution of the samples prepared under sulfur sufficient and deficient conditions are shown in Fig. 3(a). After the thin 1 nm Mo deposition, the sample is moved out of the sputtering chamber and exposed to air. The Mo film will be oxidized and form Mo oxides. During the high-temperature growth procedure, the Mo oxide segregation and the sulfurization reaction will take place simultaneously. If the background sulfur is sufficient, the sulfurization reaction will be the dominant mechanism. Most of the surface Mo oxides will be transformed into MoS_2_ in a short time. The MoS_2_ film formed on the sample surface will prevent the presence of Mo oxide segregation and coalescence. In this case, a planar MoS_2_ film will be obtained on the sapphire substrate after the sulfurization procedure. Under the sulfur deficient condition, since there is no sufficient sulfur, only limited numbers of MoS_2_ will form on the surface. In this case, Mo oxide segregation and coalescence will be the dominant mechanism at the initial stage of the sulfurization procedure. Small Mo oxide clusters are then formed on the sapphire substrates. The thick Mo oxide clusters will prevent complete transformation of the Mo oxides into MoS_2_. In this case, Mo oxides covered with few-layer MoS_2_ films will be obtained after the sulfurization procedure. The supporting evidence for the proposed growth model comes from the XPS curves of the two samples measured before and after the sulfurization procedure. The XPS curve of the sample measured before sulfurization is shown in Fig. 3(b). As shown in the figure, Mo^6+^ 3d peaks located at ~235.9 and 232.8 eV and Mo^4+^ 3d peak located at ~229.7 eV are observed. The results indicate that Mo oxides are formed before the sulfurization procedure^23^. The XPS curves measured after the sulfurization procedure for the two samples grown under sulfur sufficient (1.5 g sulfur, blue curve) and deficient (no sulfur supply, red curve) conditions are shown in Fig. 3(c). For the sample grown under the sulfur sufficient condition, the peaks representing Mo^4+^ 3d and S^2−^ 2p orbitals are observed. Due to the spin-orbit interaction, the Mo^4+^ 3d orbital splits into Mo^4+^ 3d_5/2_ and Mo^4+^ 3d_3/2_ orbitals with binding energies 229.8 and 233 eV, respectively. Similarly, the S^2−^ 2p orbital splits into S^2−^ 2p_3/2_ and S^2−^ 2p_1/2_ orbitals with binding energies 162.5 and 163.5 eV, respectively^24^. The results suggest that all the Mo oxides have been transformed into MoS_2_. However, for the sample prepared under the sulfur deficient condition, beside the Mo^4+^ 3d and S^2−^ 2p peaks, an additional Mo^6+^ 3d peak is also observed. The phenomenon indicates the co-existence of both MoS_2_ and Mo oxides after the sulfurization procedure for the sample grown under the sulfur deficient condition. The results have confirmed that depending on the amount of background sulfur, either the Mo oxide segregation or the planar MoS_2_ growth will become the dominant mechanism during the sulfurization procedure.

### Layer number dependence of MoS_2_ on pre-deposited Mo thicknesses and MoS_2_ transistors

**Figure 4:**
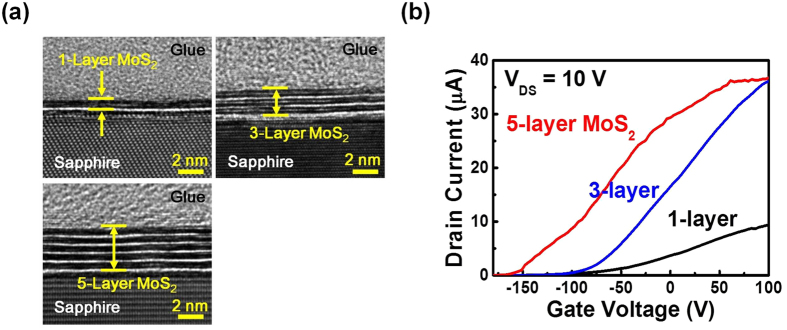
(**a**) The cross-sectional HRTEM images of the three samples with pre-deposited 0.2, 0.5 and 1.0 nm Mo films. 1-, 3- and 5- layers of MoS_2_ are obtained for the three samples. (**b**) The I_D_-V_GS_ curves of the MoS_2_ transistors fabricated by using the three samples measured at V_DS_ = 10 V.

Layer number controllability is one important issue for the growth of 2D crystals. In previous discussions, it has been predicted that the layer numbers of MoS_2_ should be determined by the Mo film thicknesses under the sulfur sufficient condition. To verify this point, two additional samples with 0.2, and 0.5 nm Mo films were prepared and sulfurized under the same sulfur sufficient condition (1.5 g sulfur powder). The cross-sectional HRTEM images of the three samples with pre-deposited 0.2, 0.5 and 1.0 nm Mo films are shown in Fig. 4(a). As shown in the figure, 1-, 3- and 5- layer MoS_2_ films proportional to the Mo film thicknesses are obtained for the three samples. The results have confirmed our previous prediction that the MoS_2_ layer number is determined by the pre-deposited Mo film thicknesses. The results have also demonstrated that a precise layer number controllability can be achieved by controlling the pre-deposited Mo film thickness. Besides the layer number controllability of this growth technique, for transistor applications, the other important issue is the control of drain current level of the fabricated devices. If similar current transport capacity can be obtained for each MoS_2_ layer prepared by using this method, it is possible to determine the required layer number of MoS_2_ transistors by simply adding up the drain current value obtained from a single-layer MoS_2_ transistor to meet the required drain current level. To verify this point, bottom-gate MoS_2_ transistors are fabricated by transferring the 1-, 3- and 5- layer MoS_2_ films to 300 nm SiO_2_/Si substrates with pre-deposited Au/Ti electrodes^13^. The I_D_-V_GS_ curves of the three devices at V_DS_ = 10 V are shown in Fig. 4(b). The drain currents for the three device at V_GS_ = 50 V are 7, 27 and 35 μA, respectively. The drain currents in positive dependence on the layer numbers of the MoS_2_ transistors have demonstrated small variation in material characteristics between each MoS_2_ layer prepared by using this growth technique. The results suggest that a better control over the drain current levels may be achieved by controlling the MoS_2_ layer numbers after further growth optimization of this growth technique in the future.

### The establishment of 2D crystal hetero-structures

**Figure 5:**
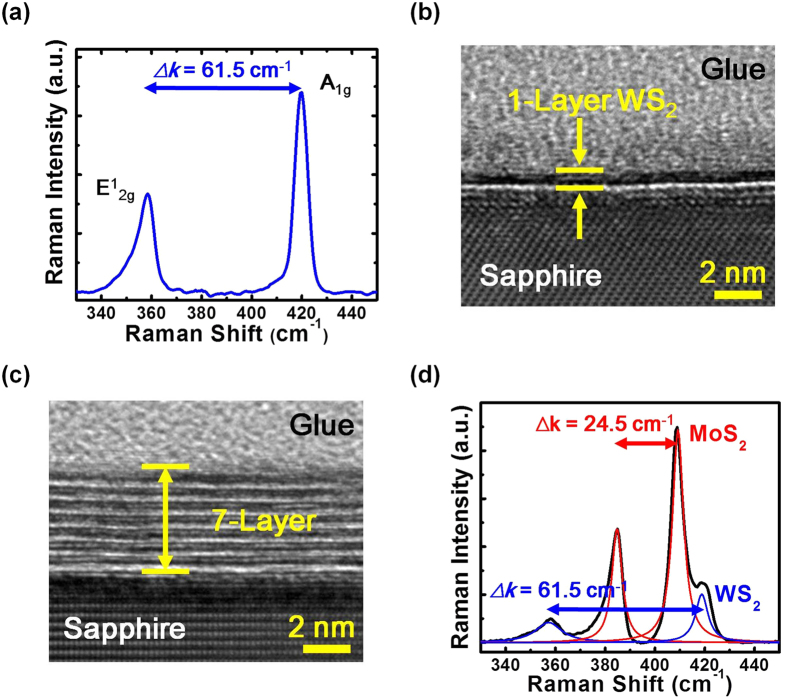
(**a**) The Raman spectrum and (**b**) the cross-sectional HRTEM image of the WS_2_ sample. (**c**) The cross-sectional HRTEM image and (**d**) the Raman spectrum of the 1-layer WS_2_/5-layer MoS_2_/1-layer WS_2_ double hetero-structure. The fitted characteristic Raman peaks corresponding to the two 2D crystals WS_2_ and MoS_2_ are also shown in the figure.

The major advantage of TMD growth by sulfurizing pre-deposited transition metals is the possibility of the hetero-structure establishment through similar growth procedures^25^,^26^. Following the same growth procedure of MoS_2_, a WS_2_ film is grown after the sulfurization of 0.5 nm pre-deposited tungsten (W) film on a sapphire substrate. The Raman spectrum of the sample is shown in Fig. 5(a). Similar with MoS_2_, two characteristic Raman peaks are observed for WS_2_, which correspond to in-plane 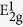 and out-of-plane A_1g_ phonon vibration modes of the WS_2_ crystal, respectively. The frequency difference *∆k* of the two Raman peaks for the WS_2_ sample is 61.50 cm^−1^, which is much larger than the *∆k* value 24.50 cm^−1^ of MoS_2_. Therefore, it is difficult to predicate the actual layer number of WS_2_ simply through the Raman spectrum. The cross-sectional HRTEM image of the sample is shown in Fig. 5(b). 1-layer WS_2_ film is obtained on the sapphire substrate. By sequential depositions of 0.5 nmW, 1.0 nm Mo and 0.5 nmW followed by the same sulfurization procedure after each metal deposition procedure, a WS_2_/MoS_2_/WS_2_ double hetero-structure can be established. Since the layer numbers for WS_2_ and MoS_2_ are 1 and 5, respectively, the total layer number for the hetero-structure should be 7. The cross-sectional HRTEM image of the sample with the hetero-structure is shown in Fig. 5(c). As shown in the figure, 7-layer WS_2_/MoS_2_/WS_2_ double hetero-structure is obtained. The results suggest that by sequential metal deposition and the same sulfurization procedures, TMD hetero-structures can be established. The identical layer number of the hetero-structure with the summation of each 2D crystal layer numbers has confirmed the excellent layer number controllability of this growth method. The other supporting evidence for the establishment of the 2D crystal hetero-structure comes from the Raman spectrum of the sample shown in Fig. 5(d). The characteristic Raman peaks corresponding to WS_2_ and MoS_2_, respectively, are observed in the figure. Compared with the standalone samples, the same Raman peak differences 24.5 and 61.5 cm^−1^ for MoS_2_ and WS_2_ suggest that the same layer numbers are obtained for the two different 2D crystals in the WS_2_/MoS_2_/WS_2_ double hetero-structure.

## Conclusion

In summary, we have demonstrated large-area and uniform MoS_2_ growth by using sulfurization of thin Mo films pre-deposited on sapphire substrates. Precise layer number controllability and the possibility of selective growth of this technique have provided an alternate choice for the growth of large-area and uniform MoS_2_. We have also proposed a growth model based on the results obtained from the sample grown under the sulfur deficient condition. Depending on the amounts of background sulfur, the competition between the Mo oxide segregation and the sulfurization reaction is the main mechanism responsible for the growth of either Mo oxide clusters covered with few-layer MoS_2_ or planar MoS_2_ films after the sulfurization procedure. The positive dependence of drain currents with increasing layer numbers of the 1-, 3- and 5- layer MoS_2_ transistors have demonstrated a small variation in material characteristics between each MoS_2_ layer prepared by using the sulfurization of pre-deposited Mo films. The demonstration of WS_2_/MoS_2_/WS_2_ double hetero-structure has revealed the advantage of this growth technique for the establishment of TMD hetero-structures.

## Methods

Before sulfurization, 1 nm of Mo is deposited on sapphire substrates by using a RF sputtering system. During the metal deposition procedure, the sputtering power is kept at 40 W and the background pressure is kept at 5 × 10^−3^ torr with 40 sccm Ar gas flow. The atomic force microscopy (AFM) image of the sample has revealed that a continuous and smooth film with surface roughness 0.19 nm is obtained after the 1.0 nm Mo deposition. After metal deposition, the samples are placed in the center of a hot furnace for sulfurization. Before sulfurization, the tube is pumped down to 5 × 10^−3^ torr to evacuate gas molecular such as oxygen from the environment. During the sulfurization procedure, 130 sccm Ar gas was used as carrier gas, while the furnace pressure was kept at 0.7 torr. The growth temperature for the samples was kept at 750 °C with the S powder placed on the upstream of the gas flow. The evaporation temperature for the S powder is kept at 120 °C. Three samples with different amounts of S powder 2.5, 2, and 1.5 g are prepared. An additional sample with no S powder is also prepared for comparison. The Raman spectrums are performed by using a HORIBA Jobin Yvon HR800UV Raman spectroscopy system equipped with 488 nm laser. The cross-sectional HRTEM and HAADF images are obtained by using a FEI Tecnai G2 F20 transmission electron microscopy system operated at 200 kV. The chemical bonds and compositions of the samples are studied by using a PHI VersaProbe II Scanning X-ray photoelectron spectroscopy (XPS) Microprobe.

## Additional Information

**How to cite this article**: Wu, C.-R. *et al*. The Growth Mechanism of Transition Metal Dichalcogenides by using Sulfurization of Pre-deposited Transition Metals and the 2D Crystal Hetero-structure Establishment. *Sci. Rep.* **7**, 42146; doi: 10.1038/srep42146 (2017).

**Publisher's note:** Springer Nature remains neutral with regard to jurisdictional claims in published maps and institutional affiliations.
